# Coronary Artery Calcium Score: Assessment of SYNTAX Score and Prediction of Coronary Artery Disease

**DOI:** 10.7759/cureus.12704

**Published:** 2021-01-14

**Authors:** Asma Shabbir, Sana T Virk, Jahanzeb Malik, Shabana Kausar, Talha B Nazir, Asim Javed

**Affiliations:** 1 Cardiology, Rawalpindi Institute of Cardiology, Rawalpindi, PAK; 2 Internal Medicine, Air University, Islamabad, PAK

**Keywords:** coronary artery calcium score, syntax, computed tomography angiography, coronary artery disease

## Abstract

Background

With the advent of modern imaging technologies, non-invasive assessment of the coronary system is not only possible but its complexity and plaque burden can be quantified. This study aims to determine whether calcium score on computed tomography coronary angiography (CTCA) can be associated with the complexity of coronary artery disease (CAD), which is determined by the SYNTAX score on coronary angiography, as well as to determine which cut-off value of coronary artery calcium (CAC) score can predict severe CAD in our population.

Methodology

This was a cross-sectional study conducted at the Rawalpindi Institute of Cardiology, Pakistan from January 2019 to March 2020. The calcium score of all patients with low-to-intermediate pretest probability of CAD was calculated on CTCA. All patients who had significant disease on CTCA were subjected to conventional coronary angiography and SYNTAX score was calculated, which was later used to determine the association between calcium and SYNTAX score.

Results

A total of 90 patients were included in the study. CAC and SYNTAX score were found to be positively correlated (Pearson coefficient [r] = 0.354; p = 0.001). The total CAC score with a cut-off value of 212 recognized patients with the SYNTAX score of >27. The sensitivity was 66.7% and specificity was 70.5% with an area under the curve of 0.743. The mean calcium score of patients in our study group was 223, with the maximum score of 1,216 and the minimum score of zero.

Conclusion

A CAC score greater than 212 is associated with a high SYNTAX score indicating complex disease. Only age is an independent predictor of calcium score.

## Introduction

Coronary artery disease (CAD) is one of the major causes of morbidity and mortality worldwide, and the people of the subcontinent are more susceptible to this disease [1-3]. The gold standard technique to assess the disease burden and its complexity is coronary angiography [4]. Past trials have shown that the severity and complexity of CAD can be assessed using the SYNTAX score [5]. In turn, SYNTAX score can predict major adverse cardiovascular events, which is a key factor in morbidity and mortality [6].

With the advent of modern imaging technologies, non-invasive assessment of the coronary system is not only possible but its complexity and plaque burden can be quantified [7]. Several registries and randomized trials have assessed the role of computed tomography coronary angiography (CTCA) in assessing the presence of CAD, defining its severity and complexity, possibly guiding decision-making, and potentially reducing cardiovascular events compared with the current standards [8-10]. This is of relevant clinical significance in busy tertiary care cardiac centers in Pakistan due to overburdened catheter labs.

A recent advancement in CTCA is the calculation of coronary artery calcium (CAC) score called the Agatston score [11]. This score is used to calculate CAC, which has been implicated in atherosclerotic plaque formation [12]. The relationship between CAC score and its quantification of future cardiac events has been demonstrated. Some studies have shown an association of CAC score with angiographically proven CAD [13,14].

The objective of this study was to determine the association of CAC with the severity and complexity of CAD on angiography with the help of the SYNTAX score, as well as to determine independent factors associated with the calcium score.

## Materials and methods

This is a cross-sectional study conducted at the Rawalpindi Institute of Cardiology Pakistan from January 2019 to March 2020. Approval was sought from the hospital ethical committee before the study (RIC/RERC/32/2020). All patients with low-to-intermediate pretest probability of CAD were included in the study. Patients with a prior history of ischemic heart disease who underwent coronary artery bypass grafting or percutaneous coronary intervention were excluded. After taking informed written consent, demographic profile was noted and detailed history was taken. Patients were examined clinically and underwent baseline workup, including renal function tests, lipid profile, and complete blood count. Patients with normal renal function tests underwent 64-multislice CTCA for assessment of significant CAD. CAC measurement was performed before CTCA in all patients. All patients were in sinus rhythm during the procedure. The total CAC score was calculated using the Vitrea 2 software. Calcium based on the Agatston score was defined as the presence of a lesion with an area greater than 1 mm^2^ and peak intensity greater than 130 HU, which was automatically identified and marked with color software. All lesions were summed up to calculate the total CAC score. Significant CAD was considered 50% or greater luminal stenosis in any major epicardial coronary artery. All patients with significant CAD on CTCA were then planned for conventional coronary angiography (CCA) after two weeks. CCA was performed by an interventional cardiologist using the Judkins technique. SYNTAX score was calculated for all patients using SYNTAX calculator software (www.syntaxscore.com). All coronary artery lesions greater than 50% on visual assessment and having a diameter greater than or equal to 1.5 mm were used to calculate SYNTAX score. The calculated SYNTAX score was divided into three tertiles as follows: less than 18 was considered low risk, between 18 and 27 was considered intermediate risk, and greater than 27 was considered high risk as per the ESC guidelines [14].

Data analysis was done using SPSS version 26 (IBM, Armonk, NY, USA). Frequency and analysis were performed for qualitative variables. Mean and standard deviations were calculated for quantitative variables. Skewness was used to check the normal distribution of continuous variables, calcium score, and SYNTAX score. Correlation of calcium score and SYNTAX score was done by Pearson’s coefficient and scatter plot analysis. Univariate and multivariate analysis along with analysis of variance (ANOVA) test was done for independent associations of calcium score. The receiver operating characteristic (ROC) curve was applied for sensitivity of high syntax for calcium scores. P-value less than 0.05 was considered significant.

## Results

The CAC score D (90) of 0.231 (p < 0.05) was significantly non-normal; hence, log 10 transformation was done and the score was labeled as CAC log. The SYNTAX score D (90) of 0.129 (p = 0.001) and skewness of 0.401 was considered to be near-normal and no transformation was done. The mean age and SD of our population was 55.79 ± 9.85 years. A total of 64 (71.1%) patients were males and 26 (28.9%) were females. The mean calcium score of patients in our study group was 223, with the maximum score of 1,216 and the minimum score of zero. A total of 17 (18.9%) patients had a calcium score of zero. The majority of patients 33 (36.7%) had a calcium score ranging 0-100. Only seven (7.8%) patients had a calcium score greater than 1,000, as shown in Table 1. The mean and SD of syntax score for patients was 15.5 ± 9.59.

**Table 1:** Frequency of patients and their calcium and SYNTAX score.

Scores	Frequency	Percentage
Calcium score		
Zero	17	18.9
1-100	33	36.7
101-400	27	30
401-1,000	6	6.7
>1000	7	7.8
SYNTAX score		
Low <18	53	58.9
Intermediate 18-27	26	28.9
High >27	11	12.2

Correlation between CAC score and SYNTAX score

**Figure 1:**
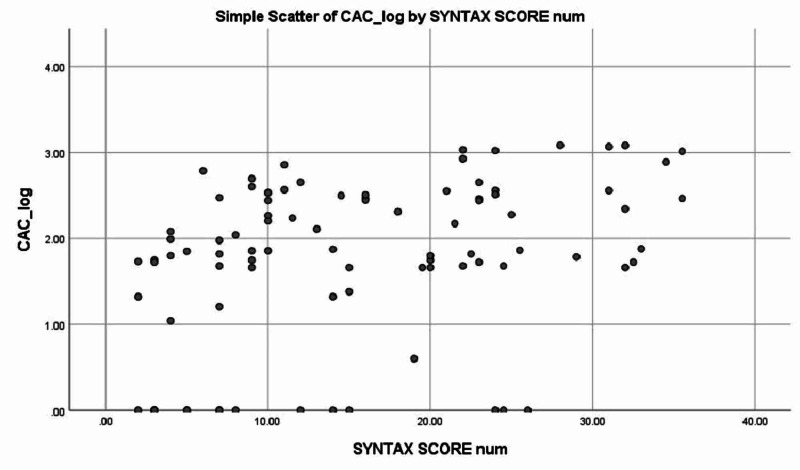
Correlation between SYNTAX and CAC score. CAC, coronary artery calcium

After checking assumptions for correlation, Pearson’s correlation analysis was performed between CAC and SYNTAX score. The two were found to be positively correlated (r = 0.354, p = 0.001). None of the independent associates were significantly correlated with the total CAC score except age (r = 0.215, p = 0.02). The other factors included diabetes mellitus (r = 0.00, p = 0.5), hypertension (r = 0.132, p = 0.10), smoking (r = 0.09, p = 0.1), and dyslipidemia (r = -0.007, p = 0.4). Figure 1 shows the correlation between SYNTAX and CAC score. Table 2 shows the SYNTAX score with different CAC scores.

**Table 2:** SYNTAX score in different groups of calcium scores.

Calcium group	SYNTAX score					
	Low <18		Intermediate 18-27		High >27	
	Count	Row N %	Count	Row N %	Count	Row N %
Zero	13	76.5%	3	17.6%	1	5.9%
1-100	20	60.6%	10	30.3%	3	9.1%
101-400	16	59.3%	8	29.6%	3	11.1%
401-1,000	4	66.7%	2	33.3%	0	0.0%
>1,000	0	0.0%	3	42.9%	4	57.1%

Independent associates of CAC score

The univariate analysis showed that age is the only predictor that was significantly associated with a higher total CAC score (p = 0.03). The rest of the predictors were not significantly associated with a higher total CAC score.

After checking the assumptions for independent variables, multiple linear regression analysis was performed to find the independent associates of the total CAC score. The model was found to be a fair predictor of CAC score (R2 = 0.178, p = 0.02). The ANOVA also showed significant linearity in line of regression (p = 0.02). Age (β = 0.208, p = 0.05) and SYNTAX score (β = 0.35, p = 0.004) were found to be significant independent predictors of total CAC score. The association of other variables are shown in Table 3.

**Table 3:** Independent associates of calcium score by univariate analysis. DM, diabetes mellitus; HTN, hypertension

Variables	Standardized β regression coefficients	P-Value
Age	0.208	0.05
Gender	0.072	0.56
DM	-0.063	0.55
HTN	-0.013	0.9
Smoking	-0.021	0.86
Dyslipidemia	0.057	0.5
SYNTAX score	0.356	0.004

**Figure 2:**
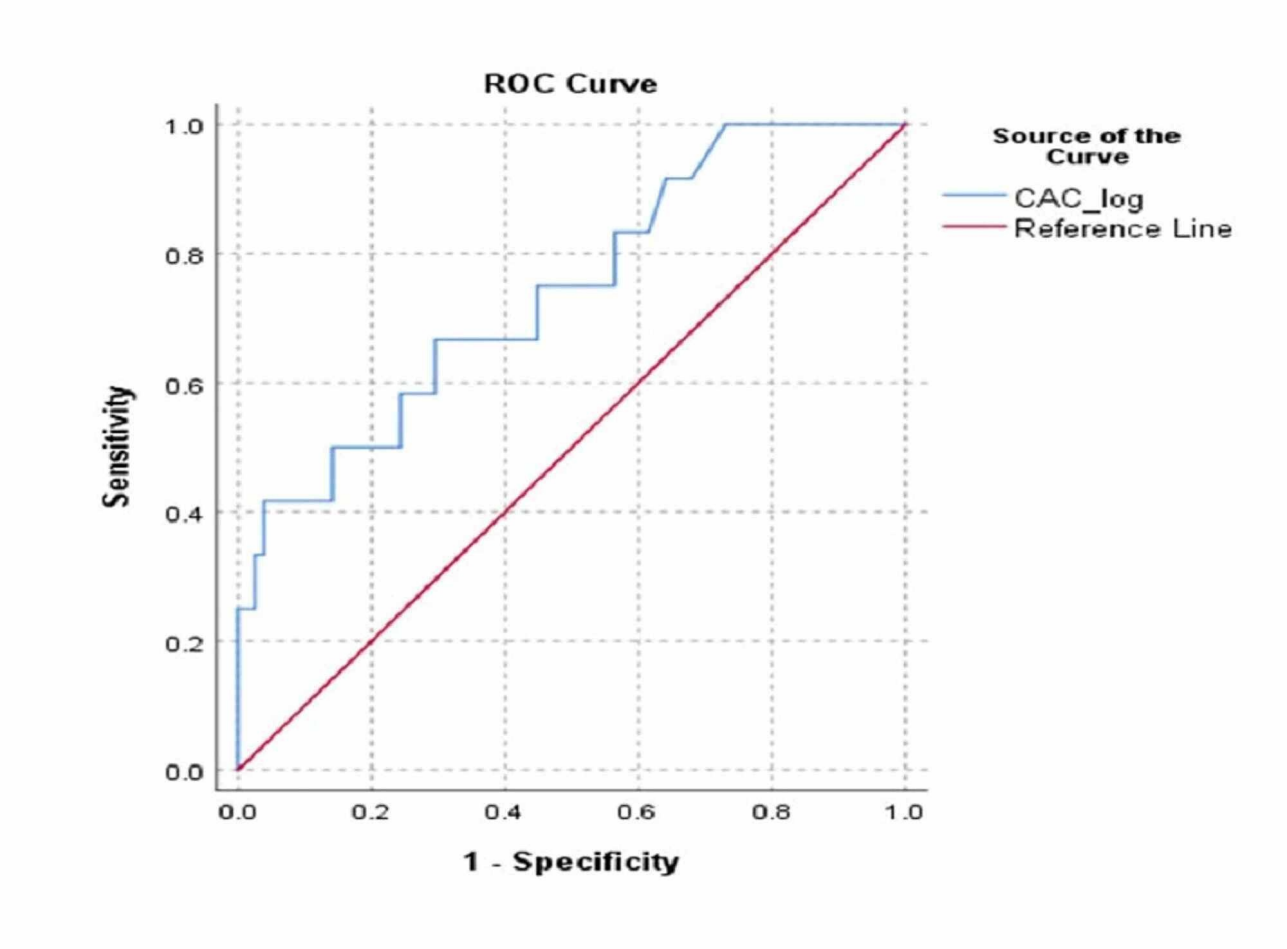
ROC curve analysis. ROC, receiver operating characteristic

The ROC curve analysis was performed to find the cut-off of total CAC score in predicting patients with SYNTAX score of >27. The total CAC score recognized patients with SYNTAX score of >27 with sensitivity of 66.7% and specificity of 70.5% with a cut-off value of 212 (area under the curve [AUC] = 0.743, 95% confidence interval [CI] = 0.589-0.896, p = 0.007). The Youden Index J statistic was found to be 0.372 for the cut-off level. The ROC curve is shown in Figure 2.

## Discussion

CAC score has been considered as an indicator of coronary atherosclerosis. Calcium score is strongly associated with coronary artery calcification and luminal stenosis [15]. It is a reliable tool for calculating the risk of myocardial infarction, coronary death, and all-cause mortality, and is also used to assess asymptomatic patients [16].

Using a registry of 25,753 asymptomatic patients who had CAC scans done were followed for long-term mortality, a study demonstrated that the number of coronaries with CAC score of >100, location of CAC, and several calcific lesions indicated coronary events [16]. According to a study conducted by Berman et al., the CAC score of zero indicates a low risk of cardiovascular disease at 15 years and generally favors a more conservative approach for management. However, if the CAC score is greater than 400, the risk is similar to that of patients having clinically significant CAD, and aggressive medical management is generally required [17]. In our study, 17 (18.8%) patients had a calcium score greater than 400 and 7 (7.78%) had a score more than 1,000. Among patients with a calcium score greater than 400, 30.8% had a low SYNTAX score on angiography and 69.2% patients had intermediate-to-high SYNTAX score.

According to another study, the risk of coronary events was 60% in patients with low-to-intermediate Framingham risk score. If these patients had a CAC score of greater than 300, the annual risk of myocardial infarction or coronary death was 2.8% and the risk at 10 years was 28%, placing these patients in the high-risk group [18]. Asymptomatic individuals, especially smokers and those with a low HDL cholesterol and low CAC score, can have moderate or severe CAD as smoking has a strong correlation with CAD [19]. However, calcium score has been recommended as a screening tool for asymptomatic patients who are at an intermediate risk of CAD as per the Framingham criteria. A study conducted in Ghana showed patients with a calcium score of more than 400 are at a greater risk of developing CAD, while those with a calcium score between 1 and 400 have twice the risk of developing CAD compared to patients with a zero calcium score [20]. A study conducted in the United States suggested there is a considerable difference in CAC based on ethnicity with Caucasians having higher calcium compared to African Americans [21]. African males were found to have higher CAC levels in contrast to females. Moreover, coronary artery calcification was seen to steadily increase with age [20]. In our study, age is the only predictor that was significantly associated with a higher total CAC score (p = 0.03).

A study conducted by Hegde and Rajendran showed that high levels of coronary calcium correlated strongly with the global coronary plaque burden, which is obtained by the SYNTAX score. Similarly, high calcium score predicts significant coronary stenosis at both individual and arterial levels [22]. In our study, there was a moderate positive association of CAC with SYNTAX score.

Different studies have reported that SYNTAX score is associated with increased cardiovascular mortality as well as difficulty in treatment. Calculation of SYNTAX score combines several morphological features of coronary artery lesions, such as chronic total occlusion, bifurcation, trifurcation, tortuosity, heavily calcified, length of lesion, aorto-ostial, and diffuse arterial lesions. In this study, 53 (58.9%) patients had a low SYNTAX score, 26 (28.9%) had an intermediate SYNTAX score, and 11 (12.2%) patients had a high SYNTAX score.

A study done by Gökdeniz et al. indicated that cut-off value of CAC greater than 809 with sensitivity of 67.6% and specificity of 87.6% could be used to predict high SYNTAX score in patients [13]. However, in our study, the cut-off of the total CAC score in predicting patients with SYNTAX score of >27 was 212 (AUC = 0.743, 95% CI = 0.589-0.896, p = 0.007). The total CAC score recognized patients with SYNTAX score of >27 with a sensitivity of 66.7% and specificity of 70.5%. The Youden Index J statistic was found to be 0.372.

## Conclusions

CAC score has a positive association with SYNTAX score and can be used to predict the complexity of CAD in patients. Therefore, all patients with low-to-intermediate pretest probability of CAD undergoing cardiac CT scan for disease assessment should have calcium score calculated, and those with a score greater than 212 in the presence of significant disease are likely to have a high SYNTAX score on coronary angiography. In our study, only age is an independent predictor of calcium score in our population.
